# Ethical and research governance approval across Europe: Experiences from three European palliative care studies

**DOI:** 10.1177/0269216320908774

**Published:** 2020-03-18

**Authors:** Nancy Preston, Johannes JM van Delden, Francesca Ingravallo, Sean Hughes, Jeroen Hasselaar, Agnes van der Heide, Lieve Van den Block, Lesley Dunleavy, Marieke Groot, Agnes Csikos, Sheila Payne

**Affiliations:** 1 International Observatory on End of Life Care, Lancaster University, Lancaster, UK; 2 Universitair Medisch Centrum Utrecht, Utrecht, The Netherlands; 3 University of Bologna, Bologna, Italy; 4 Radboud University, Nijmegen, The Netherlands; 5 Erasmus MC, Rotterdam, The Netherlands; 6 VUB-UGhent End-of-Life Care Research Group, Vrije Universiteit Brussel (VUB), Brussels, Belgium; 7 Institute of Primary Care, University of Pécs Medical School, Pécs, Hungary

**Keywords:** Ethics, surveys and questionnaires, Europe, palliative care, clinical trial as topic, research governance

## Abstract

**Background:**

Research requires high-quality ethical and governance scrutiny and approval. However, when research is conducted across different countries, this can cause challenges due to the differing ethico-legal framework requirements of ethical boards. There is no specific guidance for research which does not involve non-medicinal products.

**Aim:**

To describe and address differences in ethical and research governance procedures applied by research ethics committees for non-pharmaceutical palliative care studies including adult participants in collaborative European studies.

**Design:**

An online survey analysed using descriptive statistics.

**Setting/participants:**

Eighteen principal investigators in 11 countries conducting one of three European-funded studies.

**Results:**

There was variation in practice including whether ethical approval was required. The time to gain full approvals differed with the United Kingdom having governance procedures that took the longest time. Written consent was not required in all countries nor were data safety monitoring committees for trials. There were additional differences in relation to other data management issues.

**Conclusion:**

Researchers need to take the differences in research approval procedures into account when planning studies. Future research is needed to establish European-wide recommendations for policy and practice that dovetail ethical procedures and enhance transnational research collaborations.

**What is already known about the topic?**

- Variation in ethical review practice is recognised but mainly from the ethical committees’ perspective.
- Little evidence on the experience of gaining ethical approval for multi-site, transnational research in practice.

**What this paper adds?**

- Variation continues to exist in ethical and research governance approval procedures across Europe.
- Interpretation of General Data Protection Regulation (GDPR) differs across Europe which has implications about how research is conducted.
- Variation in practice was not related to the perceived vulnerability of patients in palliative care trials rather standard procedures applied in most countries.

**Implications for practice, theory or policy**

- Researchers need to be aware of differences in research governance procedures when planning international research in particular time to gain full approval.
- The differences in approval procedures need greater review.
- Future policy development is recommended to guide research ethical and governance procedures across Europe.

## Background

To ensure that research is ethically robust, ethical and governance review is required to carefully examine projects before approval. These procedures can be organised at national or local levels but little is known about the varying degrees of practice both within and between countries. Ethical examination requires assessment of the potential risk a research study may have. When research involves potentially vulnerable participants such as in palliative care, this scrutiny becomes even more important.

For researchers to conduct international collaborative research, there needs to be an appreciation of ethical and governance requirements in the countries involved. International variation in ethical review has been noted in the experiences of ethical review boards^1–6^ and the researchers’ perspective.^7^ While the Declaration of Helsinki promotes a set of ethical standards,^8^ in practice committees, sometimes even on a regional level, apply their own interpretation of these criteria in their approval processes. This can result in disparity with some criteria being more stringently applied than others, particularly when the research participants are receiving palliative care and perceived as vulnerable.^9–11^

Ethics committees also assess how researchers approach and access data about potential participants. The introduction of the European-wide General Data Protection Regulations^12^ will also affect these processes. Data restrictions may include access to medical records leaving identification of potential research participants to clinical staff who may not prioritise research. At the same time, gaining Good Clinical Practice certification or its equivalent is a mandatory requirement in some countries enabling more robust research practice.

## Aim

To describe and address differences in ethical and research governance procedures applied by research ethics committees for non-pharmaceutical palliative care collaborative European studies including adult participants.

## Methods

An online descriptive study was conducted to describe and compare the ethical and review governance procedures across three European studies (Seventh Framework Programme) (Box 1) with data collected in 11 different countries. Study designs included a cluster randomised controlled trial in oncological hospital settings (ACTION), a cluster randomised controlled trial in nursing and care homes (PACE) and organisational studies in community settings (InSup-C). For each study, a single research protocol was used across all partner nations to obtain ethical approval.

**Box 1.** Study characteristics.

**Advance Care Planning; An Innovative Palliative Care Intervention to Improve Quality of Life in Cancer Patients – a Multi-Centre Cluster Randomized Clinical Trial (ACTION ISRCTN63110516)** http://www.action-acp.eu Cluster randomised trial of an Advance Care Planning intervention in lung cancer and advanced colorectal cancer. Questionnaire-based study with patients and carers. Six nations: The Netherlands (lead), Belgium, Denmark, Italy, Slovenia and the United Kingdom
**Palliative Care for Older People in Care and Nursing Homes in Europe (PACE ISRCTN14741671)** http://www.eupace.eu/ Cluster randomised trial of the PACE Steps to Success Programme, a Palliative Care intervention for nursing homes. Seven nations: Belgium (lead), Finland, Italy, The Netherlands, Switzerland, Poland and the United Kingdom
**Patient-Centred Palliative Care Pathways in Advanced Cancer and Chronic Disease InSup-C** http://www.insup-c.eu/ Embedded case study examining the integration of palliative care in community-based patients with advanced cancer, chronic obstructive pulmonary disease (COPD) and heart failure. Longitudinal interviews and questionnaires with patients, carers and healthcare staff. Five nations: The Netherlands (lead), Belgium, Germany, Hungary and the United Kingdom

## Design and data collection

An online survey was used to capture the experiences. The survey was developed through consultation between the research teams and piloted on two study-naïve researchers. Ethical approval was obtained from Lancaster University (FHMREC14107). A link to the online survey was sent to 18 principal investigators in 11 countries conducting one of the three studies (responses stored on password-protected computers). The principal investigator either completed the survey or delegated it to another member of their research team. The survey comprised 20 tick box and free text domains and a narrative ‘general comments’ section. It covered questions about local ethical and governance approval procedures including necessity for ethical approval, research governance processes, timeframes, additional approvals, access to patient records, consent and Good Clinical Practice requirements. Data were analysed using descriptive statistics and content analysis.

## Results

Seventeen investigators responded (no response from Poland). Ethical approval was not required in Denmark (ACTION) and in the Netherlands (InSup-C) as the studies were not seen as trials in the same way as a drug study. In Germany (InSup-C), ethical approval was only required for patient participation rather than family carer or health care staff.

Eight of the 17 investigators were required to go through formal ethical review in addition to other approval processes. Only the United Kingdom required this on PACE. Only investigators in the United Kingdom were invited to attend the ethics committee meeting. Changes to the protocol were required post-ethical review in Belgium (InSup-C), Finland (PACE), Italy (ACTION and PACE) and the United Kingdom (PACE). The time taken to gain ethical approval varied as did additional research governance requirements (Table 1). The PACE study gained the speediest approval and ACTION the longest. Overall, the United Kingdom had the slowest approval processes.

**Table 1.** Time taken from ethical and research governance approval submission to final approval to collect data for each study/country (*n* = 17).

Months	Action	InSup-C^a^	PACE
<1		The Netherlands	Finland The Netherlands
1–3	Denmark^b^ Slovenia	Hungary	Belgium Switzerland United Kingdom
3–6	Belgium Italy	United Kingdom	Italy
6–9		Germany	
>12	United Kingdom The Netherlands		

No response from Poland.

a Missing data Belgium (InSup-C).

b Ethical approval was not required as not a trial of a medicinal product, but additional approval was gained to screen medical records from the Danish Health and Medicines Authority.

Verbal consent was seen as sufficient for interview studies in some countries such as Germany and the Netherlands in InSup-C. Where ethical approval was not required for a study (e.g. ACTION in Denmark) or certain participants such as carers or staff in InSup-C in Germany and the Netherlands, the teams chose to implement high levels of ethical practice including gaining consent.

A formal data safety monitoring committee was only mandatory in the United Kingdom (ACTION) which, in turn, led to this being required in all participating nations. However, in the Netherlands, it was stated that as ‘. . . our study was classified as low risk, we don’t need a whole data monitoring committee, but just a person who monitors our data collection process’. Good Clinical Practice certification was needed in only four countries (Table 2). In Denmark where no ethical approval was required, additional approval was gained to screen medical records from the Danish Health and Medicines Authority.

**Table 2.** Consent and good clinical practice (GCP) requirements (*n* = 17).

	Action	InSup-C^a^	PACE
GCP required	Belgium The Netherlands United Kingdom	The Netherlands United Kingdom	The Netherlands United Kingdom Switzerland
GCP not required	Denmark Italy Slovenia	Germany Hungary	Belgium Finland Italy

No response from Poland.

a Missing data Belgium (InSup-C).

Researchers were permitted to screen for potential participants by reviewing clinical notes in four sites: Finland (PACE), Hungary (InSup-C), Italy (ACTION only) and Slovenia (ACTION). Research nurses performed this function in the United Kingdom (ACTION and InSup-C), Denmark (ACTION), Finland (PACE), Italy (PACE), Switzerland (PACE) and Slovenia (ACTION). The United Kingdom was alone in needing public and patient consultation (PPI) in research design to gain approval.

## Discussion/conclusion

The value of collaborative transnational research programmes is paramount. This study aimed at providing, for the first time, palliative care researchers with a comparative view of practice in ethical and governance approval processes in 11 European Countries. The main findings were (1) considerable diversity in ethical review practice between the participating countries exists; (2) the study settings and design may influence the speed of approval decisions; and (3) overall, the United Kingdom had the longest ethical and research governance approval procedures.

As with all surveys, the responses given may not be a true representation of what happened in practice. There was some missing data, which may have skewed the results. However, nearly all responses were returned while the studies were ongoing; hence, lapses in memory were likely to be minimal. Although members of the research team completed the survey, ultimately the authors were in essence describing their own experiences, which could cause some bias.

The study corroborates and expands on previous findings reported for both observational^5,6,13^ and interventional studies^7^ in other research fields or non-trial research about palliative care.^2^ Differences still exist in ethical and research governance approval processes across Europe, especially in relation to how long studies take to be approved and what ethical committees classify as research, which need their approval. These results can help to inform researchers planning international studies by focusing attention on the variability of ethics and governance procedures for particular research designs, processes for screening potential participants, the involvement of the public in establishing research priorities and developing studies, and data management. Moreover, our results may help to increase attention to the need for agreed standards for approval procedures for non-pharmaceutical European studies, at least through training of ethical committees, as is happening in pharmaceutical studies.

Ideally, such an agreement should also include different standards for observational and interventional designs. The two randomised controlled trials, PACE and ACTION, underwent the shortest and the longest approval procedure, respectively. The longer time required for the approval of the ACTION trial may be longer because the study involved patients receiving an intervention rather than training staff to deliver an intervention, such as in PACE, and the patient group seen as more vulnerable.

Finally, considering the United Kingdom was the first Country to lead European Union FP5 and FP6 projects,^14^ the longest approval in United Kingdom is somewhat concerning. Indeed, prolonged ethical approval processes, especially when they concern the research lead country, increase research costs, delay recruitment and can slow collaborative endeavour. On the other hand, high levels of scrutiny are needed to promote ethically conducted research, especially when research involves vulnerable subjects. A balance between the ethical need to reduce the length of approving procedures, in order to make research results promptly available to the public, and the need for a careful ethical examination should be achieved.

Future research is therefore required in all of these areas to establish Europe-wide recommendations for policy and practice that dovetail ethical procedures and enhance transnational research collaborations. Indeed, even acknowledging that would be difficult to reach an international standardised approach due to different national legal frameworks concerning human research,^6^ any effort to obtain this goal is needed. The differences in ethical review are remarkable given all countries want researchers to adhere to the Declaration of Helsinki, which states in guideline 1 that it applies to (all) medical research involving human subjects, including research on identifiable human material and data.^8^ In reality, however, countries are trying to find ways to get to a risk-adaptive governance structure for the ethical review of research. Our results point to the necessity to harmonise the ethical review of this kind of low-risk research across Europe.

## Supplemental Material

Survey_questions – Supplemental material for Ethical and research governance approval across Europe: Experiences from three European palliative care studies Click here for additional data file. Supplemental material, Survey_questions for Ethical and research governance approval across Europe: Experiences from three European palliative care studies by Nancy Preston, Johannes JM van Delden, Francesca Ingravallo, Sean Hughes, Jeroen Hasselaar, Agnes van der Heide, Lieve Van den Block, Lesley Dunleavy, Marieke Groot, Agnes Csikos and Sheila Payne in Palliative Medicine
