# Supplementary material for: Ethical and research governance approval across Europe: Experiences from three European palliative care studies
Source: Palliat Med. 2020 Mar 18;34(6):817–21. doi: 10.1177/0269216320908774 (PMC7521003; doi:10.1177/0269216320908774)
Supplement: Survey_questions – Supplemental material for Ethical and research governance approval across Europe: Experiences from three European palliative care studies [file Survey_questions.docx]

Gaining ethical and research governance procedures - v1

We are grateful that you are willing to take this survey. The survey is asking about your experiences of gaining ethical and research governance procedures for PACE, InSup-C or ACTION studies. We recognise that each country has different approvals to follow and we want to gain an understanding of the approvals required for the three different types of study. In some countries you need approval from both ethics committees and from clinical settings (research governance). These can be linked but require separate approval systems. 

Q1 What country are you from?

▼ Belgium ... United Kingdom

Q2 Which study are you completing the survey on behalf of?

- InSup-C (Case Study)
- ACTION (Cluster Trial)
- PACE (Cluster Trial only - not survey)

Q3 Do you have separate ethics and clinical setting (research governance) approvals?

- Yes No Other (*please specify*)

Q4  What level of ethical approval did you require for participants who were ***patients/residents***?  (*Please tick all that apply*)

- Ethics committee approval
- Chair of ethics committee approval only
- Hospital and/or other clinical setting ethics committee approval
- University ethics committee approval
- No ethical approval required
- Not applicable

Q4a Any comments to clarify

Q5 What level of ethical approval did you require for participants who were **carers**? 
(*Please tick all that apply*)

- Full ethics committee approval
- Chair of ethics committee approval only
- Hospital and/or other clinical setting ethics committee approval
- University ethics committee approval
- No ethical approval required
- Not applicable

Q5a Any comments

Q6 What level of ethical approval did you require for participants who were **health care staff**?
(*Please tick all that apply*)

- Full ethics committee approval
- Chair of ethics committee approval only
- Hospital and/or other clinical setting ethics committee approval
- University ethics committee approval
- No ethical approval required
- Not applicable

Q6a Any comments

Q7 What type of clinical research ethics committee did you need approval from?
(*Please tick all that apply*)

- National
- Regional
- Hospital and/or other clinical setting
- Other (*please specify*) ________________________________________________

Q8 Did you attend the ethics committee meeting?

- Yes No Not applicable

Q9 Were you required to gain input from patient or public groups into the design of the study?

- Yes No Don't know

Q9a If yes, please specify

Q10 Did the ethics committee require you to make any additional changes to the agreed protocol? If so please specify:

Q11 If you required ethical approval from more than one committee please describe the order you had to follow to obtain the necessary approvals to start the study, for example, did you need approval from clinical settings before or after research ethical approval? Did you require university approval before or after clinical ethical approval etc.

Q12 From the first submission of the ethics application (to ethics committee/clinical organisation) to the full approval to recruit the first participant, approximately how long did this process take (this could have involved more than one committee)?

- < 1 month
- 1-3 months
- 3-6 months
- 6-9 months
- 9-12 months
- >12 months

Q13 Approximately how long did each stage of this process take? Please describe:

Q14  If amendments were made to the study after the initial ethics committee(s) approval how long did it take to process the amendments so you were able to implement the changes?

Q15 Are the academic research team allowed to search clinical patient records to identify potential participants?

- Yes No Don't know

Q15a Any comments

Q16 Did the ethical/governance requirements in **YOUR** country require the setting up of a data safety monitoring committee/board?

- Yes No Don't know

Q16a Any comments

Q17 Were you required to alter any of the study materials in any way to fit your country's ethical requirements, including:

- Protocol
- Participant information sheet
- Consent form
- Anything else (*please specify*)

Q17a If yes, please could you give details:

Q18 What level of consent were patients required to give?

- Written
- Verbal
- No consent required
- Not applicable

Q18a Any comments

Q19 If applicable, what level of consent were **carers** required to give?

- Written
- Verbal
- No consent required
- Not applicable

Q20 If applicable, what level of consent were **health or social care staff** required to give?

- Written
- Verbal
- No consent required
- Not applicable

Q21 Did your research staff need Good Clinical Practice (GCP) research training?

- Yes No Don't know

Q21a Any comments

Q22 Do you have access to research nurses or associates outside of the university/clinical team?

- Yes No Don't know

Q22a Any comments

Q23 When we designed this survey we were required to send information about the survey to our University Ethics Committee. Would you have been required to gain ethics approval to conduct this survey in your country?

- Yes No Don't know

Q23a Any comments

Q24 Finally, please add any other comments you may wish to share with us:
